# Autophagy Dually Induced by AMP Surplus and Oxidative Stress Enhances Hemocyte Survival and Bactericidal Capacity via AMPK Pathway in *Crassostrea hongkongensis*

**DOI:** 10.3389/fcell.2020.00411

**Published:** 2020-06-03

**Authors:** Xin Dang, Nai-Kei Wong, Yongli Xie, Vengatesen Thiyagarajan, Fan Mao, Xiangyu Zhang, Yue Lin, Zhiming Xiang, Jun Li, Shu Xiao, Zohaib Noor, Yuanqiu He, Yang Zhang, Ziniu Yu

**Affiliations:** ^1^CAS Key Laboratory of Tropical Marine Bio-resources and Ecology, Guangdong Provincial Key Laboratory of Applied Marine Biology, South China Sea Institute of Oceanology, Chinese Academy of Sciences, Guangzhou, China; ^2^The Swire Institute of Marine Sciences, School of Biological Sciences, The University of Hong Kong, Hong Kong, China; ^3^Southern Marine Science and Engineering Guangdong Laboratory, Guangzhou, China; ^4^National Clinical Research Center for Infectious Diseases, Shenzhen Third People’s Hospital, The Second Hospital Affiliated to Southern University of Science and Technology, Shenzhen, China; ^5^Innovation Academy of South China Sea Ecology and Environmental Engineering, Chinese Academy of Sciences, Guangzhou, China

**Keywords:** AMPK phosphorylation, apoptosis, autophagy, Hong Kong oyster, infection, ROS, *Vibrio parahaemolyticus*

## Abstract

*Crassostrea hongkongensis* (Hong Kong oyster) is an ecologically and economically valuable shellfish endemic to South/Southeast Asia. Due to ocean acidification and warming waters, they have become increasingly vulnerable to invading microbes including *Vibrio parahaemolyticus*, a significant foodborne human pathogen. In recent years, outbreaks of *V. parahaemolyticus* have emerged as a perennial phenomenon in parts of the world, necessitating to better understand the biology of host-pathogen interactions in this under-examined marine invertebrate. Although an immunologically relevant autophagy apparatus has been identified in *Crassostrea gigas*, an evolutionarily close mollusk cousin, the precise mechanistic details of *C. hongkongensis* autophagy during *V. parahaemolyticus* infection are still wanting. Here, we compellingly demonstrated that *in vivo V. parahaemolyticus* challenge robustly triggered autophagic signaling in *C. hongkongensis* hemocytes peaking at 6 h post-infection, which subsequently promoted bacterial clearance and dampened premature apoptosis. Simultaneously, a large surplus of adenosine monophosphate (AMP) and elevations in reactive oxygen species (ROS, specifically mitochondrial O_2_^–^ and cellular H_2_O_2_) formation were observed post-infection. Extrinsically applied AMP and ROS could synergistically induce AMP-activated protein kinase (AMPK) phosphorylation to stimulate downstream autophagic events. *V. parahaemolyticus* infection-induced autophagy was pharmacologically shown to be AMPK-dependent *in vivo*. Overall, our results establish autophagy as a crucial arm of host defense against *Vibrio* infections in mollusks, and provide new insights into the underappreciated roles of ROS and AMP as co-regulators of autophagy.

## Introduction

Climate change and anthropogenic degradation of coastal ecosystems in the past decades have a progressive and profound impact on pathogen distribution in marine hosts consumed within the human food chain (Baker-Austin et al., 2012; Vezzulli et al., 2016). A case in point is the Hong Kong oyster (*Crassostrea hongkongensis*), an edible mollusk species with an aquaculture history of 700 years and an annual production exceeding 1.6 million tons (Lam and Morton, 2003). A sessile filter feeder of intertidal zones, *C. hongkongensis* subsists on filtering seawater replete with microorganisms and possesses an immune system that has co-evolved with several clinically significant marine pathogens. Among these, *Vibrio* spp. including *Vibrio parahaemolyticus* (*V.p*.) have been implicated as a major cause of mortality and morbidity through seafood-associated gastroenteritis worldwide (World Health Organization and Food and Agriculture Organization of the United Nations, 2011). Notably, coastal regions of the Pacific basin are increasingly prone to foodborne diseases caused by *V. parahaemolyticus* outbreaks (Nordstrom et al., 2007; Cheng et al., 2013; Wu et al., 2014). As a result of ocean acidification and warming waters, encroachment of *V. parahaemolyticus* is accelerated in shellfish harvesting areas, forcing some aquaculture industries to shut down (Marsha et al., 2018; Richards et al., 2019).

Although devoid of adaptive immunity like other marine invertebrates, *C. hongkongensis* has evolved a sophisticated innate immune system to cope with assaults from diverse biotic and abiotic agents, including bacterial and viral infections (Zhang et al., 2015; Wang et al., 2017). Hemocytes in these oysters play pivotal roles in defining both the cellular and humoral arms of innate immunity, via regulated processes like phagocytosis and generation of antimicrobial reactive oxygen species (ROS) (Wang et al., 2017; Zhou et al., 2018). Pathogen clearance typically involves immune recognition, intracellular signal transduction and downstream effector activation leading to eradication exogenous pathogens such as *V. parahaemolyticus* (Chen et al., 2018; Mao et al., 2018). Recently, carbamazepine-induced macroautophagy in hemocytes of the Pacific oyster (*Crassostrea gigas*) was found to present autophagic features similar to mammalian cells (Picot et al., 2019). However, our understanding on the functional significance and mechanistic details of autophagy in bacterial infections in oysters is far from complete.

Autophagy is a highly regulated destructive mechanism to disassemble dysfunctional cytoplasmic components and injected pathogens (Klionsky et al., 2016). As best characterized in eukaryotic cells, the autophagic machinery is coordinately regulated by multiple autophagy-related genes (Atg) and other signal transducers in response to diverse stressful conditions including nutrient deprivation, oxidative stress and microbial infections (Mizushima et al., 2011). In invertebrates, homologs of Atg exist in conserved pathways among diverse species, including *Caenorhabditis elegans*, *Drosophila melanogaster*, *Apis mellifera*, and *Ciona intestinalis* etc. (Navajas et al., 2008; Chera et al., 2009; Nelly et al., 2009; Zhang et al., 2013; Nagy et al., 2015). As part of a cellular antimicrobial system, autophagy serves to eliminate invading microbes *en masse*, in order to promote homeostasis and survival of the host cells (Vojo et al., 2013; Kewei, 2015). In marine invertebrates, the immunological roles of autophagy are not well understood though it has been shown to be cytoprotective in *C. gigas* against infections by the oyster-specific pathogens ostreid herpesvirus 1 (OsHV-1) and *Vibrio aestuarianus* (Pierrick et al., 2015). Activated oyster hemocytes produce ROS which signal danger, exert antimicrobial effects, and mobilize downstream pathways in immune responses (Bachère et al., 2015). Meanwhile, emerging evidence suggests that hypercatabolism or stressful conditions such as infections and severe injuries could lead to changes in energetic status characterized by amino acid deprivation and an altered AMP/ATP ratio, culminating in the phosphorylation of AMP-dependent protein kinase (AMPK) and inhibition mTOR to stimulate autophagy (Filomeni et al., 2015). Thus far, however, a gap remains in our mechanistic understanding of how oxidative stress and energy deprivation unfold in the bacterial infections in marine invertebrates and whether they act in concert to potentiate autophagic signaling.

In this study, we show that autophagy prevailed in *C. hongkongensis* hemocytes which became potentiated upon *V. parahaemolyticus* infections *in vivo*. Autophagy was essential for clearing the invading microbes and protected hemocytes from inordinate premature apoptosis. Additionally, infection drove an accumulation of intracellular AMP and ROS, which then triggered AMPK activation synergistically in hemocytes. We have thus provided the first direct evidence that autophagy exhibits anti-apoptotic and bactericidal properties in the hemocytes of *C. hongkongensis* as an important marine invertebrate species linked to vibriosis in human.

## Materials and Methods

### Animals Acclimation, Pathogen Challenge, and Hemolymph Preparation

Two-year-old *C. hongkongensis* (shell length 100 ± 10 mm) were collected from Zhanjiang, Guangdong Province, China, and acclimated to laboratory conditions by rearing in aerated sand-filtered seawater at ambient temperature (24 ± 1°C) and appropriate salinity (18‰) for 2 weeks prior to experiments. During the acclimation period, oysters were fed with the microalgae *Isochrysis galbana* (10^5^ cells/mL) and *Chaetoceros calcitrans* (2 × 10^5^ cells/mL) twice a day.

*Vibrio parahaemolyticus* used in the study had been isolated from *C. hongkongensis* following established methods (Mao et al., 2018). For *in vivo* bacterial challenge, bacteria were cultured in LB (Luria-Bertani) broth at 37°C to reach OD_600__*nm*_ = 0.6∼0.8, and then centrifuged at 800 × *g* at 4°C for 10 min. Afterward, bacteria were washed 3 times with PBS (phosphate-buffered saline, 0.14 M sodium chloride, 3 mM potassium chloride, 8 mM disodium hydrogen phosphate dodecahydrate, 1.5 mM potassium phosphate monobasic, pH 7.4). Bacterial pellet was re-suspended in PBS to a density of OD_600__*nm*_ = 1.0. Oysters in the challenged group were injected with 100 μL bacterial suspension into the adductor muscles, while oysters in the untreated control group were injected with an equal volume of PBS. After injection, oysters were returned to separate fiberglass tanks. Subsequently, hemolymph was withdrawn from the pericardial cavity through adductor muscles of *C. hongkongensis* by means of a sterile 1-mL syringe with an 18 G1/2-inch needle. Hemolymph was kept on ice to prevent cellular aggregation or clotting and centrifuged immediately at 200 × *g* for 10 min to harvest hemocytes. The supernatant was passed through a 0.22-μm filter paper, as hemolymph serum. Hemocytes were maintained in hemolymph serum at 18°C in petri dishes until subsequent analyses.

### Pharmacological Intervention of Autophagy

To inhibit autophagy, oysters were treated with NH_4_Cl (Sigma-Aldrich, 09718) at 20 mM for 6 h in aquariums (3 L of seawater). For chemical induction of autophagy, oysters were injected with 200 μL carbamazepine (10 mM, diluted in dimethyl sulfoxide) into the adductor muscles, and then transferred back to aquariums for 6 h. To test the effects of exogenous AMP (Sigma-Aldrich, 01930) and H_2_O_2_ (Fluka, 216763) on autophagy induction or APMK phosphorylation, each oyster was injected with 30 mg AMP into the adductor muscles and treated with H_2_O_2_ at 10 mM for 6 h in 3L seawater. Additionally, for inhibiting the AMPK pathway, Compound C (Selleck, S7306) was injected at a dose of 20 μg individually. To validate the role of oxidative stress on autophagy induction, mito-TEMPO, as a broadly used mitochondria-specific ROS scavenger, was injected at a dose of 0.5 mg per oyster, by using the same method.

### Immunoblotting and Analysis

Hemocytes were collected from a pool of 5 individuals as biological replicates, and each experimental condition had triplicates. For every pool, hemocytes were homogenized in 100 μL cold IP lysis buffer supplemented with a protease and phosphatase inhibitor cocktail according to the manufacturer’s instructions (Sangon Biotech, C50035). Lysates were centrifuged at 1,200 × *g* for 20 min at 4°C. The supernatant was diluted 10× to determine the protein concentrations by using bicinchoninic acid (BCA) protein assay (Thermo Fisher Scientific, 23227). Lysates were mixed with a 4 × protein loading buffer (200 mM Tris, 8% sodium dodecyl sulfate, 400 mM DTT, 0.4% bromophenol blue, 40% glycerol, pH 6.8) and heated to 95°C for 15 min. Then, the homogenate containing approximately 60 μg per lane of total protein was loaded onto a 12% polyacrylamide gel for SDS-PAGE, which were subsequently transferred to 0.2 μm PVDF membranes (Merck Millipore, ISEQ00010). The membranes were blocked with QuickBlock^TM^ blocking buffer for Western blot (Beyotime, P0252) and then incubated overnight with primary antibodies at 4°C. Later, the membranes were washed with PBST (PBS with 0.1% Tween-20), and incubated for 2 h at room temperature with a 5,000 × diluted anti-rabbit HRP-linked secondary antibody (Cell Signaling Technology, 7074). After washing 3 times with PBST, membranes were visualized following reactions with an ECL luminescence reagent (Sangon Biotech, C510043). Relative protein expression was quantified by using the ImageJ software (v1.8.0). The following primary antibodies were used to detect their corresponding protein substrates: rabbit anti-LC3 (Cell Signaling Technology, LC3A/B 4108), rabbit anti-AMPKα (Cell Signaling Technology, 2603), rabbit anti-phospho-threonine 172-AMPKα (Cell Signaling Technology, 2535), and rabbit anti-β-actin (Cell Signaling Technology, 8457). The housekeeping protein β-actin was selected as a loading control, and its blotting was done within its linear range of detection (Supplementary Figure S4).

### Transmission Electron Microscopy

Hemocytes were pooled after being collected from 10 individuals per group as described above for TEM observation. Cells were fixed in an electron microscope fixation solution (Servicebio, G1102) for 4 h at 4°C. The suspension was centrifuged at 200 × *g* for 10 min and washed with a 0.1 M phosphate buffer (pH 7.4) for 3 times (15 min each). Pellets were post-fixed with 1% osmium tetroxide in 0.1 M phosphate buffer at room temperature for 2 h. After washing with the same buffer for 3 times, pellets were collected and embedded in 2% agar. Following dehydration in a graded series of ethanol and acetone, pellets were embedded in Epon 812 (SPI, 90529-77-4) and polymerized at 60°C for 48 h. Ultrathin sections of 60 nm in thickness were mounted onto a copper grid and stained with uranyl acetate and then lead citrate (15 min each). Electron micrographs were acquired by using a transmission electron microscope (Hitachi, HT7700).

### Total RNA Extraction and Quantitative Real-Time PCR Analysis

Total RNA of harvested hemocytes was isolated with TRIzol reagent (Invitrogen, 15596-026) according to the manufacturer’s instructions. RNA quality and quantity were determined and assessed by using NanoDrop 2000C (Thermo Fisher Scientific, United States). Extracted RNA was reverse-transcribed into cDNA with the PrimerScript^TM^ first strand cDNA synthesis kit (TAKARA, RR047A) and then subjected to quantification analysis by using 2 × RealStar Green Power mixture (GenStar, A311) in LightCycler^®^ 480 II (Roche, Switzerland), according to the manufacturer’s instructions. Gene-specific primers were designed with Primer Premier v5.0, and their sequences are as listed in Supplementary Table S1.

### Bacterial Clearance Assay

Prior to infection, hemocytes were plated and cultured in 24-well plate (8 × 10^5^ cells per well) for 30 min at 27°C. After harvest, three strains of live bacteria (*Escherichia coli*, *Vibrio alginolyticus*, and *V. parahaemolyticus*) were washed 3 times, resuspended in PBS, and diluted to a concentration of 8 × 10^7^ CFU/mL. To initiate infection, 10 μL of bacterial suspension was added to each well to achieve an MOI (multiplicity of infection) = 1. Following incubation for 40 min at 27°C, extracellular bacteria were removed by washing 3 times with 0.02% trypsin-EDTA. Subsequently, hemocytes were lysed and homogenized in 1 mL cold PBS containing 0.05% Triton X-100 for 1 h. Ten microliters of lysates from each well were serially diluted in PBS, from which a 100-μL bacterial inoculum was spread on LB plates, followed by overnight culture to enumerate bacterial colony counts. Negative controls were set up with hemocyte lysates unexposed to bacteria (Supplementary Figure S5).

### Flow Cytometric Analysis of Apoptosis and Mitochondrial Superoxide Formation

Hemocytes were collected and resuspended in 100 μL binding buffer, followed by incubation (10 min in dark) with 5 μL Annexin V-FITC and 5 μL propidium iodide (PI) supplied by an apoptosis detection kit (Vazyme, A211) at room temperature. Next, another 400 μL binding buffer was added to the suspension, before analysis by a Guava^®^ easyCyte^TM^ flow cytometer (Millipore). For superoxide detection, hemocytes were harvested after *in vivo* bacterial exposure to oysters. They were then resuspended in Ca^2+^ and Mg^2+^ supplemented Hank’s balanced salt solution (HBSS; BBI, E607006) with 5 μM MitoSOX reagent (Invitrogen, M36008) at 37°C for 10 min. Finally, hemocytes were washed and resuspended in fresh HBSS for detection of mitochondrial superoxide by flow cytometry. At least 10,000 gated cell events were included. Statistical analysis was performed with FlowJo (v10.0 software).

### TUNEL Assay

Hemocytes were collected and plated on 24-well plate (8 × 10^5^ cells per well) for 30 min, then were washed by PBS and fixed with 4% paraformaldehyde for 30 min at room temperature. Subsequently, hemocytes were washed by PBS again and treated with 500 μL PBS containing 0.3% Triton X-100 for 5 min, then washed by PBS for three times and stained by using One-Step TUNEL Cell Apoptosis Detection Kit (Green Fluorescence) (Beyotime, C1088) according to manufacturer’s instructions. Samples were counterstaining with DAPI (Sigma, D9564) and examined under fluorescence microscope (Thermo Fisher Scientific, EVOS).

### H_2_O_2_, AMP, and GSH/GSSG Measurement Post-infection

Hydrogen peroxide was measured by Amplex Red hydrogen peroxide/peroxidase assay kit (Invitrogen, A22188) according to the manufacturer’s instructions. Briefly, hemocytes were collected and mixed isometrically in a working solution containing 10 U/mL horseradish peroxidase and 10 mM Amplex Red. The mix was incubated at room temperature for 30 min, shielded from light. Absorbance at 560 nm was determined by using an EnSight^TM^ Multimode plate reader (PerkinElmer, United States). Quantitative measurements were obtained against a standard curve (Supplementary Figure S6A) of H_2_O_2_ consisting of a gradient of concentrations (Sigma, 88597) and then normalized with respect to total proteins. For AMP assay, oyster hemocytes were seeded into a microplate after *in vivo* infection detected, followed by measurements at 450 nm according to the instructions for human AMP ELISA kit (RenJieBio, RJ12284), which were then quantified with a standard curve (Supplementary Figure S6B). For antioxidant evaluation, GSH/GSSG content was determined at 412 nm by using a GSH and GSSG assay kit (Beyotime, S0053) according to the manufacturer’s instructions, which was then quantified with a standard curve (Supplementary Figures S3A,B).

### Statistical Analysis

Data processing and statistical analyses were performed by using GraphPad Prism (v8.0.1), and a heatmap was generated by R (v3.5.2). Comparisons between two groups of samples were performed by Student’s *t*-test and those among more than two groups by one-way ANOVA, followed by Tukey’s or Dunn’s *post hoc* tests by SPSS (v22.0).

## Results

### *V. parahaemolyticus* Infection Induced Autophagy in *C. hongkongensis* Hemocytes

**FIGURE 1:**
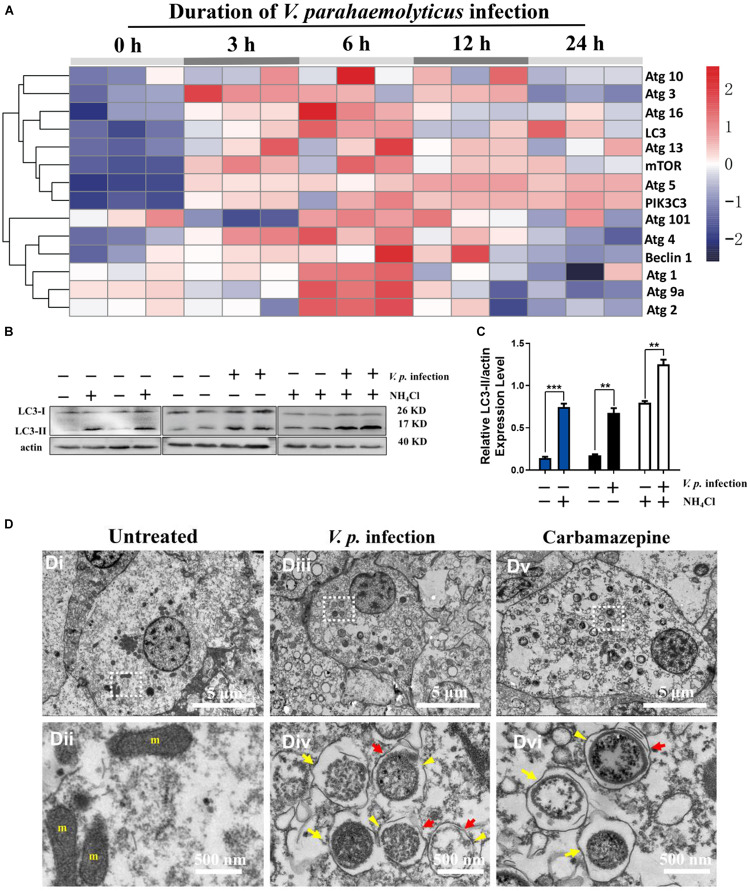
*V. parahaemolyticus* infection induces autophagy in hemocytes of the Hong Kong oyster. **(A)** Heatmap showing the profile of autophagy-related genes expressed in *C. hongkongensis* hemocytes at 0, 3, 6, 12, and 24 h after *V.p.* infection as detected by quantitative real-time PCR with GAPDH as an internal control. Data are scaled by *z* score (*n* = 3 biological replicates). **(B)** Western blot for LC3 expression *in vivo* in hemocytes before and after bacterial infection (for 6 h), with NH_4_Cl as an autophagy inhibitor. **(C)** Ratio of LC3-II to β-actin in densitometric analysis by Student’s *t*-test between two groups. Data are presented as mean ± SEM (*n* = 3), with statistical significance determined at **p < 0.01, ***p < 0.001. **(D)** Ultrastructural morphology of autophagic vacuoles in hemocytes by transmission electron microscopy. **(Di,ii)** Representative electron micrographs of hemocytes in resting condition (untreated). **(Dii)** Region at a higher magnification for the inset in **(Di)**, showing few autophagic vacuoles present in the cytosol. **(Diii,iv)** Representative electron micrographs of hemocytes at 6 h post-infection (*V.p*. infection). **(Div)** Region at a higher magnification for the inset in panel **(Diii)**, showing single (yellow arrow) and double (yellow arrowhead) membranes with electron-lucent clefts (red arrow). **(Dv,vi)** Representative electron micrographs of hemocytes exposed to an autophagy inducer (carbamazepine). **(Dvi)** Region at a higher magnification for the inset in panel **(Dv)**, showing single (yellow arrow) and double (yellow arrowhead) membranes with electron-lucent clefts (red arrow). m, mitochondria.

To characterize autophagy in hemocytes following bacterial infection, we set out to obtain temporally resolved profiles of mRNA expression of 14 autophagy-related genes in the hemocytes of *C. hongkongensis* challenged with *V. parahaemolyticus*. Several genes expression exalted post-challenging, which reached peak levels at 6 h (Figure 1A). Accordingly, we picked this time point for observation in subsequent experiments. Changes in LC3-I and LC3-II levels were observed by Western blot, following treatment with the autophagy inhibitor NH_4_Cl to test the functional integrity of autophagic pathway in *C. hongkongensis* hemocytes. As expected, NH_4_Cl treatment increased the LC3-II to actin ratio compared to that in the untreated control (Figures 1B,C), accompanied by an accumulation of autophagic vacuoles in hemocytes (Supplementary Figure S1). On the other hand, *V.p.* infection evidently induced LC3-II accumulation, which could be further enhanced by NH_4_Cl treatment, suggesting that autophagy had been triggered by *V.p.* infection in hemocytes (Figures 1B,C). The appearance of global autophagic vacuoles was confirmed in TEM (transmission electron microscopy), which are characterized by both single- (yellow arrow) and double-membranes (yellow arrowhead) with an electron-lucent cleft (red arrow) in the cytoplasm. These features were compared to corresponding ultrastructures and morphology of autophagic vacuoles elicited by the established autophagy inducer carbamazepine (Figure 1D). The results suggest that autophagic flux was present in *C. hongkongensis* hemocytes and it culminated at 6 h after infection of *V. parahaemolyticus.*

### Autophagy Contributed to Bacteria Clearance in Hemocytes

**FIGURE 2:**
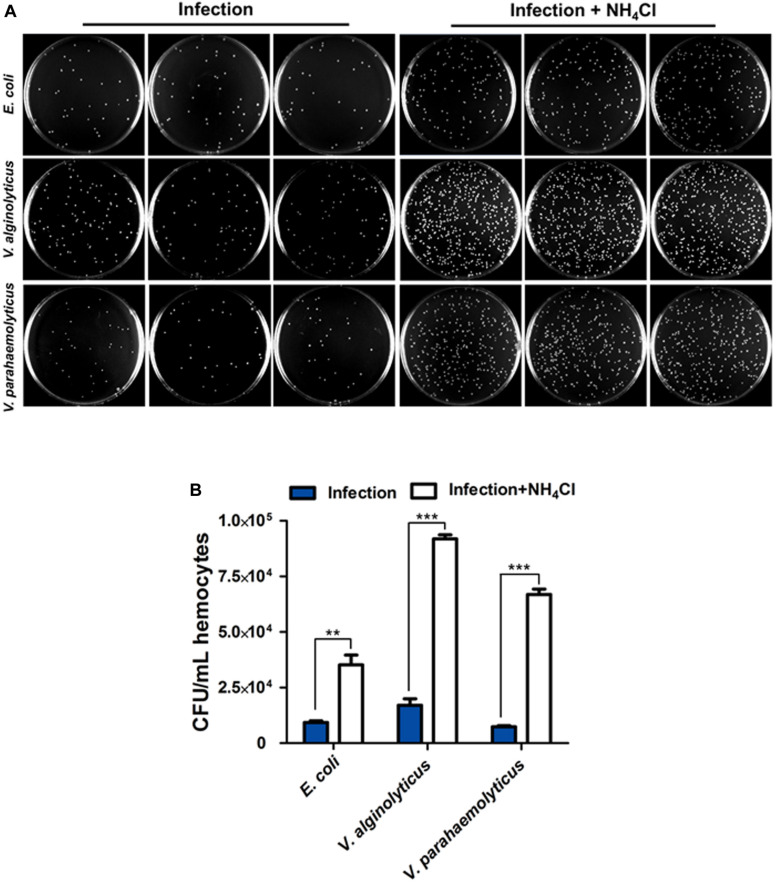
Autophagy contributes to bacteria clearance in hemocytes. **(A)** Representative agar plating results for infection outcomes with or without treatment of NH_4_Cl as an autophagy blocker. *C. hongkongensis* hemocytes were exposed to *Escherichia coli* (*E. coli*), *Vibrio alginolyticus* (*V. alginolyticus*) or *Vibrio parahaemolyticus* (*V. parahaemolyticus*). **(B)** Statistical difference in the extent of bacteria clearance the control and NH_4_Cl groups was determined by Student’s *t*-test between. Enumeration of bacterial colonies was presented as mean ± SEM (*n* = 3 plates), with significance being determined at **p < 0.01 and ***p < 0.001. Results are representative of three independent experiments.

Bacterial clearance assay was performed to ascertain the roles of autophagy in host defense against bacteria. Following inhibition of autophagy with NH_4_Cl, the colony forming units (CFU) of three strains including *Escherichia coli*, *Vibrio alginolyticus*, *V. parahaemolyticus* were dramatically increased approximately by 4 fold, 5 fold and 9 fold, respectively, with respect to the untreated control (Figures 2A,B), suggesting that autophagy promoted antibacterial activity in hemocytes.

### Autophagy Protected Hemocytes From Premature Apoptosis During Bacterial Infection

**FIGURE 3:**
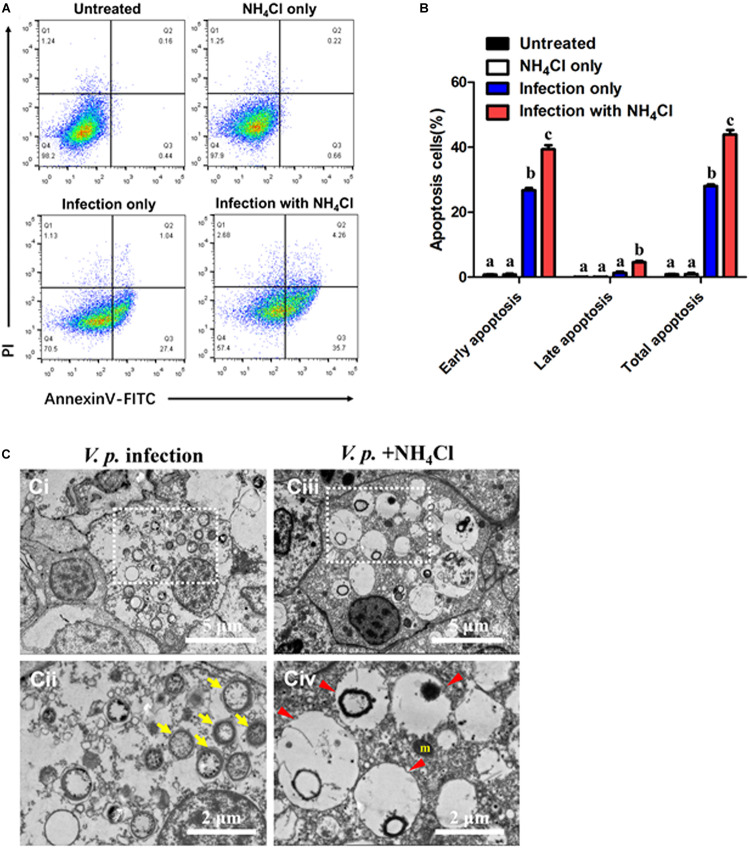
Autophagy prevents premature apoptosis in hemocytes post-infection. **(A)** Representative dot-plot results in flow cytometry (FACS) for apoptosis (6 h) in hemocytes before (up panels) and after infection (down panels), with the autophagy blocker NH_4_Cl as an intervention. Q1: Cells stained positive with PI only (upper left quadrant) were necrotic/non-viable cells. Q2: Cells stained doubly positive by Annexin V-FITC and PI (top right quadrant) were cells undergoing late apoptosis/necrosis. Q3: On the lower right quadrant were cells stained positive by Annexin V only, representing cells undergoing early apoptosis. Q4: The lower left quadrant (PI and Annexin V negative cells) shows live cell population. **(B)** Statistical evaluation of the proportions of early apoptosis (Q3), late apoptosis (Q2) and total apoptosis (Q2 + Q3) cells by one-way ANOVA followed by Dunn’s *post hoc* test indicated by different alphabetical letters (*p* < 0.05). Data are presented as mean ± SEM (*n* = 4). **(C)** Ultrastructural morphology of hemocytes in transmission electron microscopy (TEM). **(Ci,ii)** Representative images of the hemocytes at 6 h-post-infection (*V.p*. infection). **(Cii)** Magnified image of region indicated in panel **(Ci)**, showing global autophagic vacuoles in the cytosol (yellow arrow). **(Ciii,iv)** Representative image of the hemocytes exposed to autophagy inhibitor after infection (*V.p.* + NH_4_Cl) for 6 h. **(Civ)** Higher magnification of region indicated in panel **(Ciii)**, with large vacuoles in the cytosol (red arrowhead). m, mitochondria.

Microbial pathogens are known to either induce or prevent apoptosis to augment their infectivity. In the case of phagocytes, inordinate or premature apoptosis would limit their bactericidal capacity. In this context, apoptosis assay was performed by dual staining of Annexin-V and PI during *V.p.* infection in the presence or absence NH_4_Cl treatment (Figure 3A). In resting condition, percentage of apoptotic cells (in terms of early, late or total apoptosis) had no significant difference between the untreated and NH_4_Cl groups (*p* > 0.05), indicating that inhibition of autophagy had no obvious effects on basal apoptosis (Figure 3B). Similar results were also validated by the TUNEL assay (Supplementary Figure S2). In post-infection condition, the bulk of cell population entered early apoptosis (in Q3) and was significantly larger than that in the untreated group (*p* < 0.05), which suggests stress being imposed within the infected hosts. Consistent with the observation on *V.p.* infection in Figure 2, when hemocytes were challenged with *V.p.* in the presence of NH_4_Cl, more individual hemocytes emerged fragmented DNA (Supplementary Figure S2), and the proportions of cells entering late and total apoptosis were further increased (*p* < 0.05), suggesting that hemocytes indeed failed to contain the bacteria while prematurely entering apoptosis (Figures 3A,B). Dramatic morphological alterations associated with these outcomes were confirmed in TEM. In the *V.p.* + NH_4_Cl condition, ultrastructural modifications resulting from apoptosis included numerous large transparent vacuoles in the cytoplasm (red arrowhead), swollen mitochondria and condensation of chromatins (Figure 3C). These observations reveal that during *Vibrio* infections, autophagic processes played apparently cytoprotective roles by reducing premature apoptosis and stabilizing turnover of hemocytes.

### Infection Induced AMP Surplus and AMPK Phosphorylation

**FIGURE 4:**
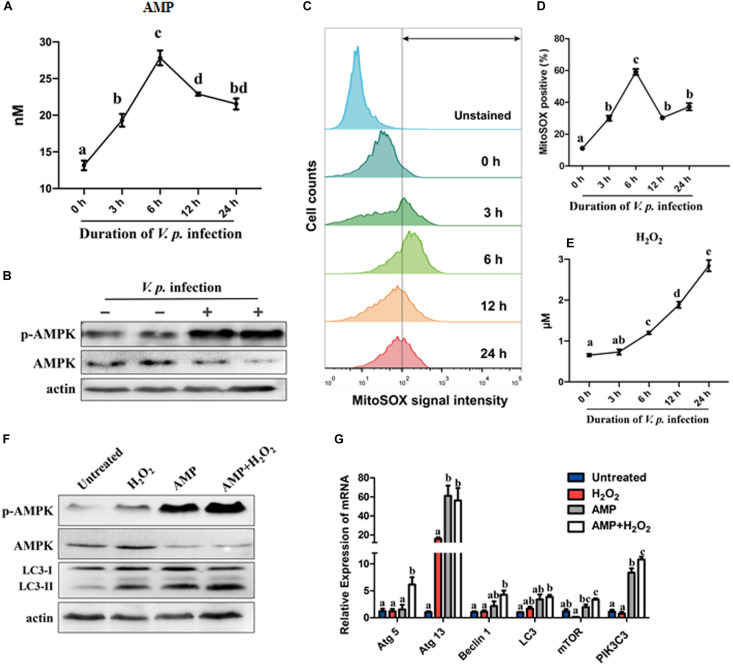
Infection-induced AMP surplus and ROS accumulation synergistically activate AMPK and autophagy signaling. **(A)** Quantification of AMP in *C. hongkongensis* hemocytes after *V.p.* challenge at 0, 3, 6, 12, and 24 h determined in ELISA for AMP and normalized by total proteins (per gram). **(B)** Western blot showing a rise in AMPK phosphorylation 6 h post-infection. **(C)** Superoxide generation in oyster hemocytes at 0, 3, 6, 12, and 24 h after bacterial challenge as detected in FACS analysis with MitoSOX as a mitochondrial superoxide probe. **(D)** Quantification of MitoSOX fluorescence intensities. **(E)** Time course study on hydrogen peroxide formation at 0, 3, 6, 12, and 24 h post-infection. Data are normalized by total proteins (per gram). **(F)** Western blot showing the synergistic effects of AMP and H_2_O_2_ on AMPK phosphorylation and autophagy induction. **(G)** Relative transcript expression of autophagy-related genes in the presence of AMP and/or H_2_O_2_. One-way ANOVA followed by Tukey’s **(A,G)** or Dunn’s **(D,E)** *post hoc* test was used to determine any significant difference among all groups, as indicated by different alphabetical letters (*p* < 0.05). Data are presented as means ± SEM (*n* = 3).

To assess cellular energy status of host cells during *V.p.* infection, the content of AMP in hemocytes was quantified following *V.p.* challenge. Basal concentration of cytosolic AMP was determined to be approximately 13 nM per gram of total protein. This began to rise upon onset of infection, and peaked at 6 h post-infection with a nearly twofold increase relative to the baseline, which temporally paralleled expression profiles of autophagy-related genes (Figures 4A, 1A). Nonetheless, AMP levels declined gently and remained at ∼20 nM up to 24 h post-infection. Furthermore, AMPK phosphorylation increased sharply while total AMPK decreased at 6 h after initial *V.p.* challenge, suggesting that *V.p.* infection drove activation of the AMPK pathway during autophagy in oyster hemocytes (Figure 4B).

### *Vibrio* Infection Promoted ROS Accumulation and Oxidative Stress in Hemocytes

Oxidative stress is one of the hallmarks of infection-induced autophagy, though the exact roles of specific ROS remain unclear. In this study, we evaluated the kinetics of formation of two major ROS in hemocytes, namely, superoxide (O_2_^–^) and H_2_O_2_. Notably, mitochondrial superoxide levels increased significantly following bacterial challenge, culminated at 6 h post-infection, and declined moderately toward 12 h and 24 h post-infection (Figures 4C,D), which broadly resembled the fluctuations in AMP levels and expression of autophagy-related genes (Figures 4A, 1A). In contrast, H_2_O_2_ levels steadily rose across the same period after infection (Figure 4E). Meanwhile, oxidative stress was evaluated by measuring and comparing GSH and GSSG levels. Upon bacterial challenge, GSH decreased and GSH/GSSG showed a falling trend, suggesting that hemocytes experienced a progressive depletion of GSH resulting in intensified oxidative stress (Supplementary Figure S3C). Collectively, these results support the notion that hemocytes were oxidatively stressed by 6 h post-infection, which was a cellular event concurrent to autophagy.

### AMP and H_2_O_2_ Synergistically Activated AMPK Pathway and Autophagy

As observed in the preceding section, AMP and H_2_O_2_ accumulated concomitantly. To clarify the links between AMP, H_2_O_2_ and autophagy as well as the functional significance therein, we treated oyster hemocytes with AMP and/or H_2_O_2_. Strikingly, both AMPK phosphorylation level and autophagy increased (Figure 4F) after AMP, H_2_O_2_ or combined (AMP + H_2_O_2_) treatment. As anticipated, the combined treatment was much more effective than treatment with single simulants. This robust response in signal transduction seems to be mirrored in the expression patterns of 6 selected autophagy-related genes (Figure 4G). Overall, elevations in AMP and H_2_O_2_ levels strongly activated autophagy in *Vibrio* infection contexts in a synergistic manner.

### Modulatory Roles of AMPK Pathway and Oxidative Stress in Autophagy Induction

**FIGURE 5:**
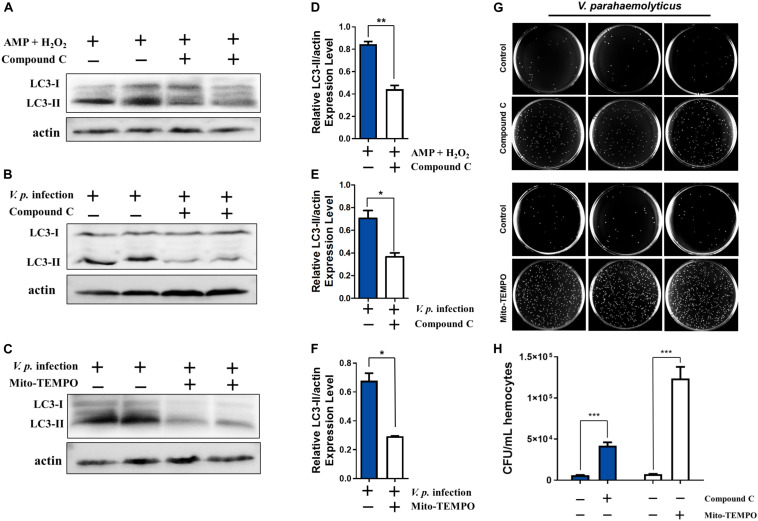
Stimulatory effects on infection induced autophagy are dependent on AMPK pathway and oxidative stress. **(A)** Western blot analysis showing that autophagy induced by AMP + H_2_O_2_ co-treatment declined after treatment with the AMPK inhibitor Compound C. **(B,C)** Western blot analysis showing that autophagy induced by *V.p.* infection (for 6 h) was blocked after treatment with Compound C or mitochondria-specific ROS scavenger mito-TEMPO. **(D)** Densitometric analysis of AMP + H_2_O_2_ co-treatment with or without Compound C on the ratio of LC3-II to β-actin by Student’s *t* test. **(E,F)** Densitometric analysis of *V.p.* infection after treatment with Compound C or mito-TEMPO on the ratio of LC3-II to β-actin by Student’s *t* test, respectively. **(G)** Representative agar plating results for infection outcomes with treatment of Compound C (up panel) and mito-TEMPO (down panel). *C. hongkongensis* hemocytes were exposed to *Vibrio parahaemolyticus* (*V. parahaemolyticus*). **(H)** Statistical difference in the extent of bacteria clearance the control and Compound C (blue bar) or mito-TEMPO (white bar) groups was determined by Student’s *t*-test between. Data was presented as mean ± SEM (*n* = 3), with significance being determined at *p < 0.05, **p < 0.01, ***p < 0.001.

**FIGURE 6:**
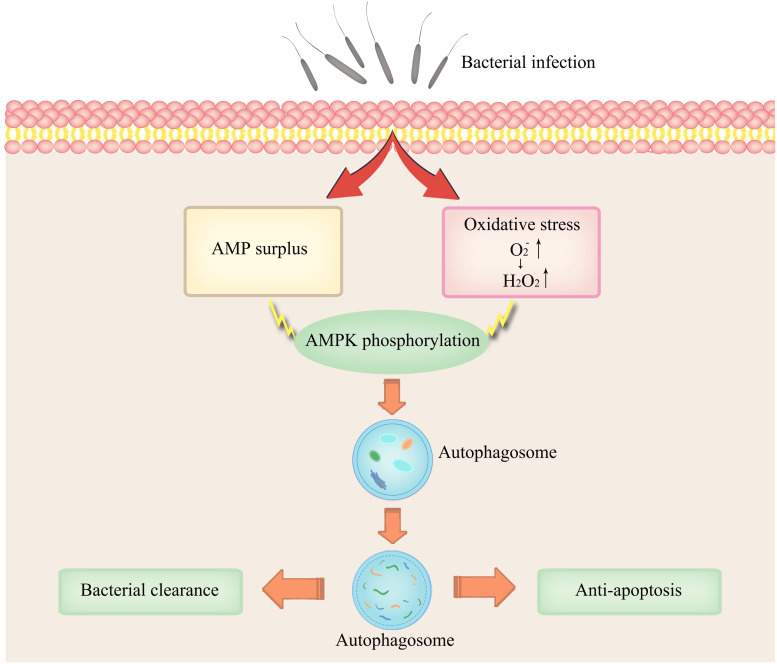
Conceptual representation of the links between AMPK activation and infection-induced autophagy synergistically induced by AMP and ROS.

As combined treatment of AMP and H_2_O_2_ further enhanced infection-induced autophagy and AMPK signaling, we attempted to clarify the roles of AMPK signal transduction in autophagy. We tested whether AMPK signaling mediated AMP + H_2_O_2_-induced autophagy by inhibiting AMPK activation with Compound C. In the presence of the AMPK inhibitor, AMP + H_2_O_2_-induced autophagy was blunted in terms of relative expression level of LC3-II, which was reduced to about half of that in the control group (Figures 5A,D). Similarly, *V.p.* infection-induced autophagy also decreased dramatically upon Compound C treatment and the corresponding relative expression level of LC3-II dropped to half of control (Figures 5B,E). Furthermore, Compound C-treated hemocytes attenuated bacterial immune defense with almost sevenfold increased CFU of *V.p.* infection *in vitro* (Figures 5G,H), confirming modulatory role of AMPK pathway in autophagy induction. Simultaneously, *V.p.* infection-induced autophagy could also be crippled upon mitochondria-specific antioxidant, mito-TEMPO treatment accompanied by halving expression of LC3-II (Figures 5C,F), indicating the regulatory role of oxidative stress in autophagy induction. Finally, the potential immunological function of oxidative stress was determined by bacterial clearance assay under treatment of mito-TEMPO, with a dramatic increasing of colony (*p* < 0.001) and a seriously flawed immune armor reasoningly (Figures 5G,H). These results suggest that AMP and ROS synergistically induced AMPK activation is likely a proximal way mediating autophagy induced by *Vibrio* infections (Figure 6).

## Discussion

Autophagy is evolutionarily conserved to a remarkable extent across species and occurs in all eukaryotic cells (Augustine et al., 2013). It is widely appreciated that autophagy operates in containing and eliminating invading microbes during infections to aid host. For immunity in invertebrates, autophagy-related natural variation reportedly raised resistance against microsporidian pathogens in *C. elegans* (Keir et al., 2019). In *Drosophila melanogaster*, autophagy was found crucial for PGRP-LE mediated autophagy induction and subsequent containment of intracellular growth of *Listeria monocytogenes* and for a PI3K-Akt mediated antiviral program against vesicular stomatitis virus (Tamaki et al., 2008; Shelly et al., 2009). Moreover, autophagic processes have been shown to work in some marine invertebrates during development, nutritional stress, microbial infections in mollusks (Pierrick et al., 2015; Balbi et al., 2018), though the molecular mechanisms remain incompletely understood. In this study, we first characterized *V.p.* infection-induced autophagy in *C. hongkongensis* by an integrated approach and used established autophagy inducers and inhibitors validated in *C. gigas* by Picot et al. (2019). In the context of *Vibrio* infections, we found that AMP-mediated energy stress and ROS stress were aggravated as infection progressed in hemocytes, which seems to play an instrumental role in autophagy.

Accumulating evidence has shown that oysters possess a highly intricate immune system molded through generations of evolutionary and competitive processes (Zhang et al., 2015). Importantly, the rate of apoptosis in hemocytes has emerged as an important parameter for evaluating the strength of immune response, though a link in immune complementarity between apoptosis and autophagy remains to be substantiated (Fitzwalter and Thorburn, 2016). In our study, our results revealed that *V.p.* infection could increase the percentage of cells undergoing early apoptosis (Figure 3A), whereas no significant difference was found in late apoptosis nor DNA fragmentation relative to the control and NH_4_Cl groups (Figure 3B and Supplementary Figure S2). Of note, only in the case of *V.p.* infection in the presence NH_4_Cl did hemocytes shift to the late apoptosis quadrant (Figure 3A) and materialize disintegrated DNA (Supplementary Figure S2). This observation supports the interpretation that although bacterial infections may stimulate hemocyte apoptosis, infections alone do not necessarily lead to eventual cell death. It is in agreement with “reversible” apoptosis first described by Hoeppner et al. (2001) in *C. elegans*, in which the cell subjected to pro-apoptosis can still potentially be rescued. In contrast, “irreversible” apoptosis induced by *V.p.* infections and NH_4_Cl was confirmed by TEM, as characterized by globally expansive, electron-lucent vacuoles in the cytoplasm (Figure 3C). As proposed by Susan Elmore, such large transparent vacuoles are a salient feature of late apoptosis, along with swollen mitochondria and electron-dense nuclei (Elmore, 2007). Remarkably, similar ultrastructural modifications were also observed by Picot et al. (2019) in hemocytes of *C. gigas* under carbamazepine (autophagy inducer) + NH_4_Cl treatment, with features resembling those in the treatment used in this study (*V.p.* infection + NH_4_Cl). Based on our findings for *V.p.* infection as a more naturalistic stimulus of autophagy (Figure 1), the molecular and cellular aspects of this complex process in marine invertebrates are substantially elaborated.

Invasion by pathogens into host organisms generally evokes immune responses as well as inflammation, which could sap host energy and disrupt cellular metabolic pathways, giving rise to disordered conditions ultimately (Cotto et al., 2019). Consistent with this, autophagy in *C. gigas* extrinsically induced by OsHV-1 viral infection, *Vibrio splendidus* infection or heat stress was characterized by accelerated energy consumption and metabolic perturbations (Corporeau et al., 2014; Zhang et al., 2019). Nutritional deprivation is one of the most studied extrinsic stressors in autophagy, typically involving amino acid or glucose imbalances in the cytosol. In this study, we have provided compelling evidence that *V.p.* infection could induce energy stress as reflected by AMP elevations and AMPK phosphorylation (Figures 4A,B). Increased cytosolic AMP content is a potent driver of cellular adaptive response to energy depletion. Changes in ATP-to-AMP ratio drives the activation of AMPK, which is conserved in *C. hongkongensis* and functionally involved in phagocytosis and cytoskeleton remodeling (He et al., 2019). AMPK activated by AMP turns off the energy-consuming pathway and induces autophagy in eukaryotic models (Dan et al., 2011), in agreement with our current data (Figure 4F). In addition, we found that the formation of superoxide and hydrogen peroxide after bacterial infection paralleled the activation of AMPK and induction of autophagy in hemocytes (Figure 4). Infection-induced ROS generation is common in marine invertebrates including oysters (Genard et al., 2013) as a means to establish a microbiologically hostile oxidizing host environment. Collaterally, oxidative stress coupled to depletion of endogenous antioxidants like GSH has been reported as a rapid inducer of autophagy (Filomeni et al., 2015). Our work illustrates that infection-elicited oxidative stress could potently enhance autophagy (Figures 5C,F), as H_2_O_2_ paired with AMP to synergistically increase AMPK phosphorylation (Figures 4F,G) and contributed to bacterial clearance (Figures 5G,H). Collectively, our results show that robust autophagic response is pivotal to *C. hongkongensis*’s ability to manage and survive bacterial assaults, thus in part accounting for the immunological resilience of mollusks. In order to illuminate the mechanistic roles and significance of energy and oxidative stress in the contexts of infection-induced autophagy, further investigation is warranted.

## Data Availability Statement

The datasets generated for this study are available on request to the corresponding author.

## Author Contributions

XD, N-KW, YZ, and ZY: conceptualization. XD, N-KW, and YZ: methodology. XD, N-KW, and YX: analysis and investigation. XD, YX, and VT: data curation. XD and N-KW: writing – original draft preparation. N-KW, FM, XZ, YL, and ZN: writing – review and editing. YX and YH: visualization. YZ and ZY: supervision. YZ, ZY, ZX, JL, and SX: project administration. YZ, ZY, ZX, and JL: funding acquisition.

## Conflict of Interest

The authors declare that the research was conducted in the absence of any commercial or financial relationships that could be construed as a potential conflict of interest.

## Abbreviations

AMP: adenosine monophosphate
AMPK: AMP-activated protein kinase
Atg: autophagy-related gene
GAPDH: glyceraldehyde 3-phosphate dehydrogenase
GSH: reduced glutathione
GSSG: glutathione disulfide
LC3: microtubule-associated protein 1 light chain 3
mTOR: mechanistic target of rapamycin (serine/threonine kinase)
PIK3C3, phosphoinositide 3-kinase: catalytic subunit type 3
ROS: reactive oxygen species
TEM: transmission electron microscopy
*V.p*.: *Vibrio parahaemolyticus.*
