# Supplementary material for: Autophagy Dually Induced by AMP Surplus and Oxidative Stress Enhances Hemocyte Survival and Bactericidal Capacity via AMPK Pathway in Crassostrea hongkongensis
Source: Front Cell Dev Biol. 2020 Jun 3;8:411. doi: 10.3389/fcell.2020.00411 (PMC7325953; doi:10.3389/fcell.2020.00411)
Supplement: Supplementary file 1 [file Data_Sheet_1.docx]

| 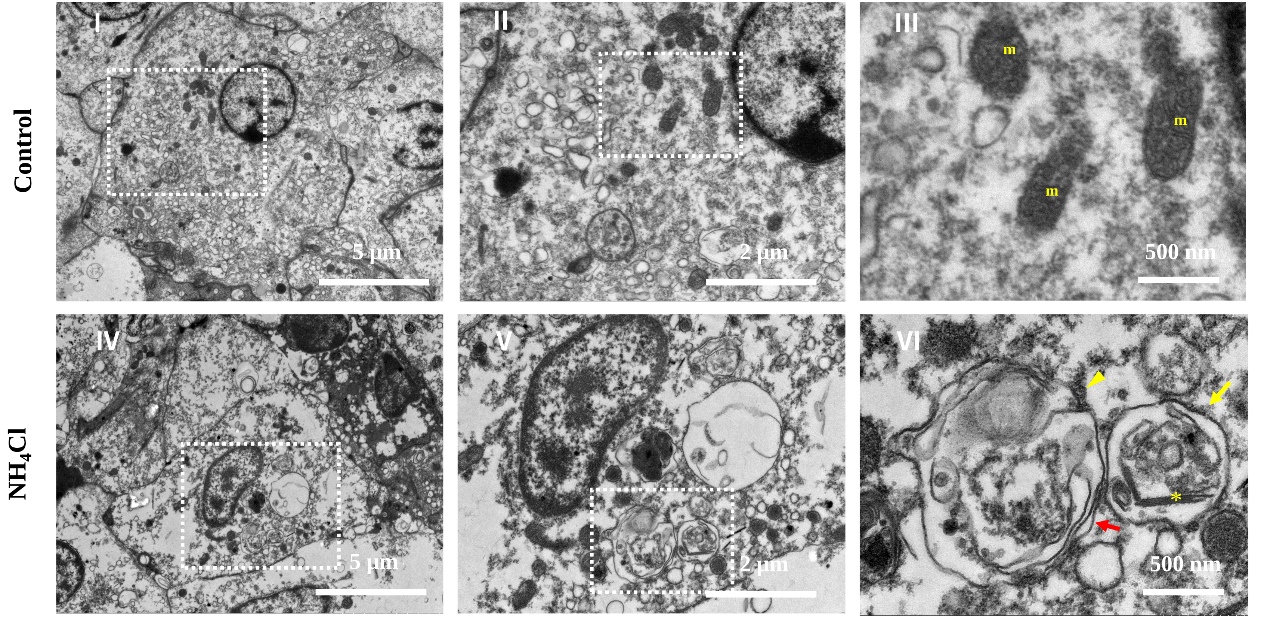 |
| --- |
|  |

**Figure S1. Ultrastructural morphology of autophagic vacuoles in *C. hongkongensis* hemocytes by transmission electron microscopy.**

I-III. Representative electron micrographs of hemocytes in resting condition (untreated). II: Region at higher magnification for inset as indicated in I. III: Region at higher magnification for inset as indicated in II. Results for this group show few autophagic vacuoles present in the cytosol. IV-VI. Representative electron micrographs of hemocytes under NH_4_Cl treatment for 6 h (NH_4_Cl). V: Region at higher magnification for inset as indicated in IV. VI: Region at higher magnification for inset as indicated in VI. Results for this group show single (yellow arrow) and double (yellow arrowhead) membranes with electron-lucent clefts (red arrow). m, mitochondrion. *, undegraded endoplasmic reticulum.


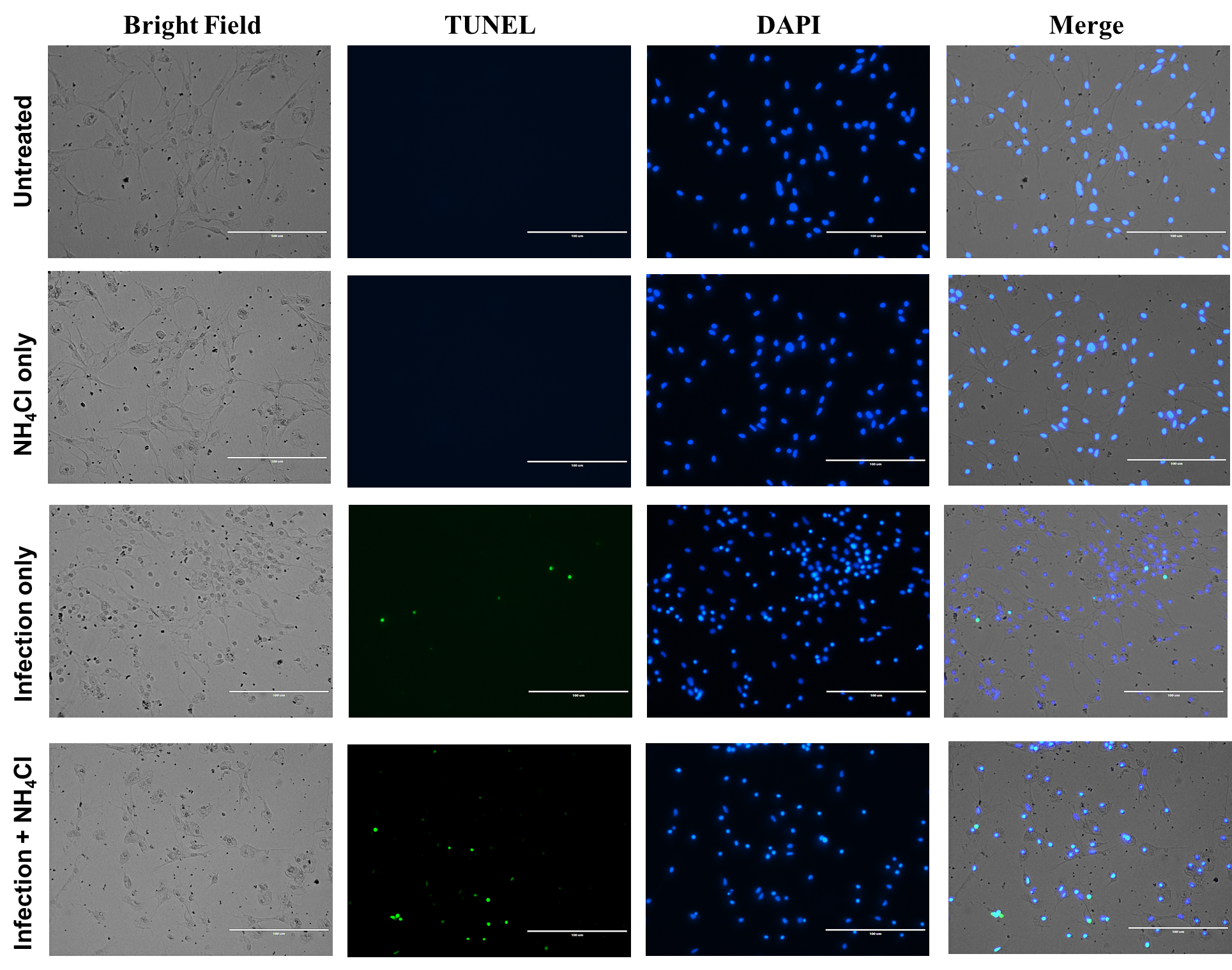


**Figure S2. Fluorescence micrographs of detection of hemocytes with fragmented DNA before and after infection with the autophagy blocker NH_4_Cl as an intervention by TUNEL assay.**

Representative fluorescence micrographs of hemocytes with FITC-staining apoptotic nuclei (TUNEL-positive) and DAPI staining of nucleic acids (blue). Scale bar, 100µm.

| 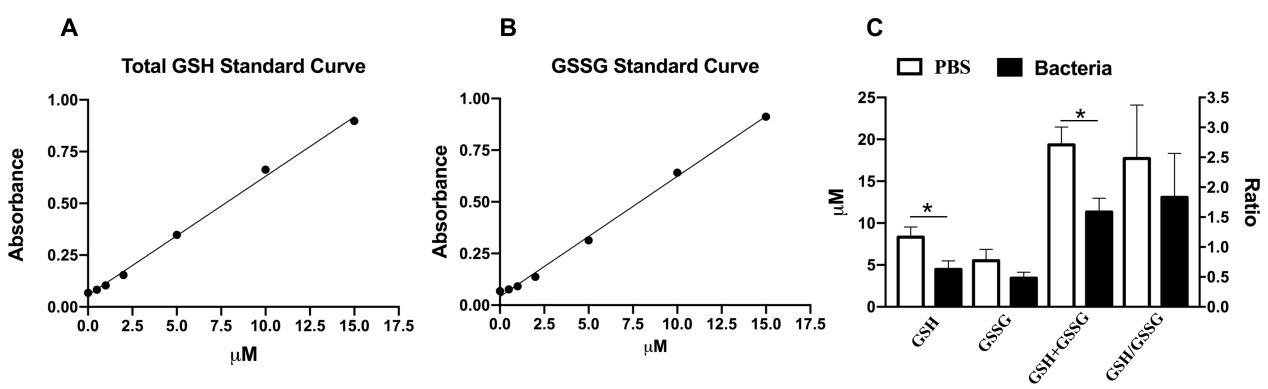 |
| --- |

**Figure S3. Total GSH and GSSG content after *V.p.* infection.**

**(A** and **B)** Standard curves for total GSH in **(A)** and GSSG in **(B)** were constructed based on absorbance measurements at 412 nm. The equations were y = 0.05746x + 0.05617 (*r^2^* = 0.9969) for GSH in **(A)** and y = 0.05822x + 0.04126 (*r^2^* = 0.9971) for GSSG in **(B)**. **(C)** Statistical analyses on GSH, GSSG, and GSH+GSSG content and the ratio of GSH to GSSG in *C. hongkongensis* hemocytes after *V.p.* infection for 6 h. Data are presented as mean ± SEM (*n* = 6), with significance being determined at **p* < 0.05.


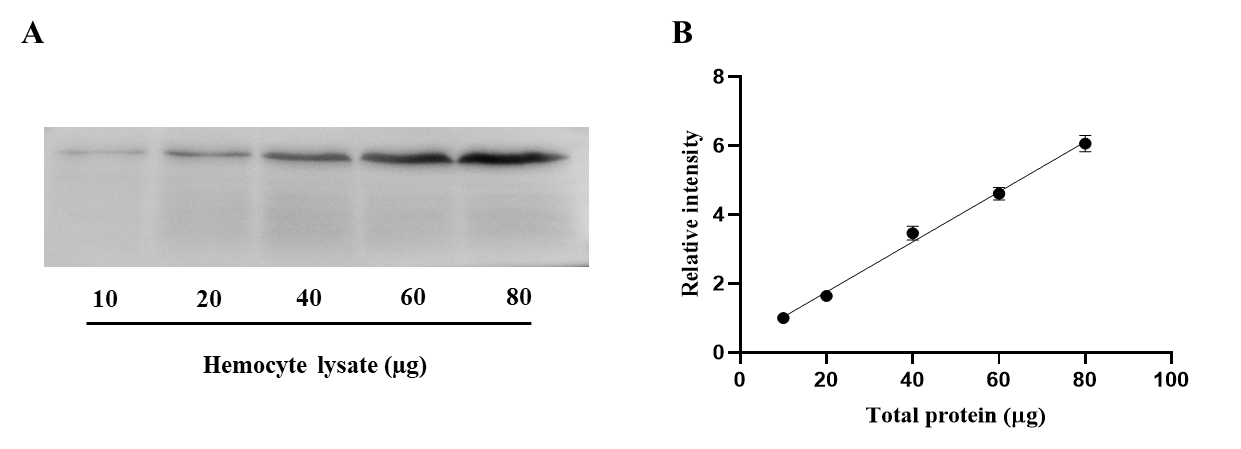


**Figure S4. Assessment of protein quantity using increasing amounts of hemocyte lysate.**

**(A)** Immunoblot of the housekeeping protein as loading control actin. **(B)** Standard curve for protein quantitation. The equation y = 0.07262x + 0.3051 (*r^2^* = 0.9902) was calculated by least-squares method with GraphPad Prism (v8.0.1).

| 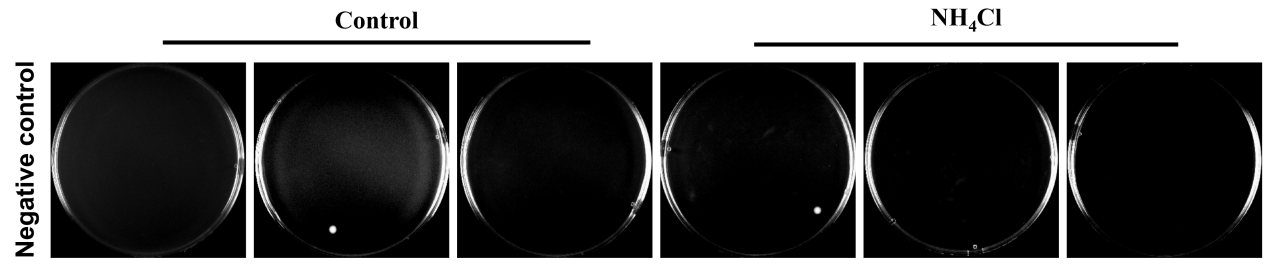 |
| --- |

**Figure S5. Negative control for bacterial clearance assay.**

As a negative control for bacterial clearance assay, *C. hongkongensis* hemocytes were collected with or without NH_4_Cl treatment prior to exposure to bacteria, which shows that few pre-existing bacteria were present in the unchallenged hemocytes, even with the inhibition of autophagy.

|  |
| --- |

**
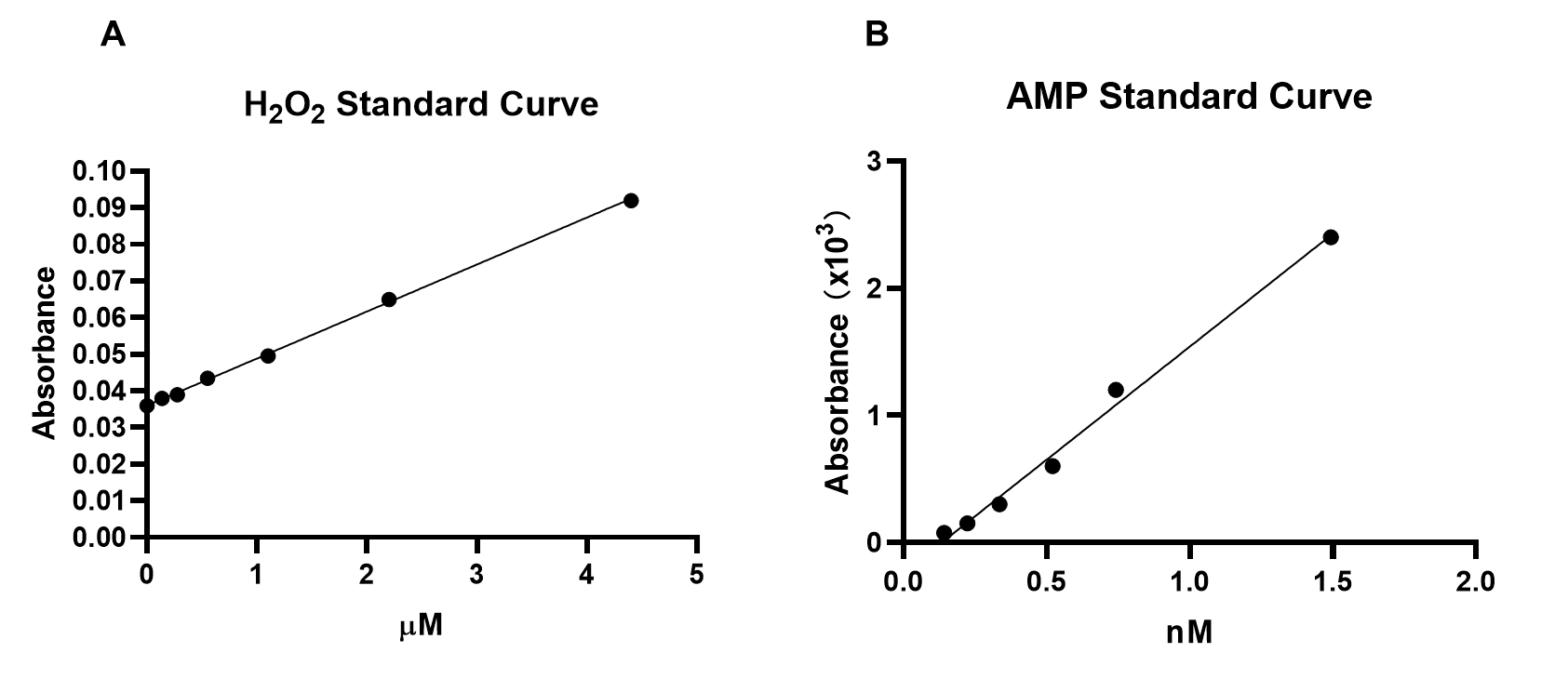
**

**Figure S6.** **Standard curves for** **H_2_O_2_ and AMP**.

**(A** and **B)** Standard curves were constructed with absorbance measurements at 560nm for H_2_O_2_ in **(A)** and 450 nm for AMP in **(B)**, normalized by amounts of total proteins determined by bicinchoninic acid (BCA) protein assay. The equation y = 0.01284x + 0.03601 (*r^2^* = 0.9981) in **(A)** and y = 1.778x - 0.2366 (*r^2^* = 0.9926) in **(B)** were calculated by least squares method with GraphPad Prism (v8.0.1).


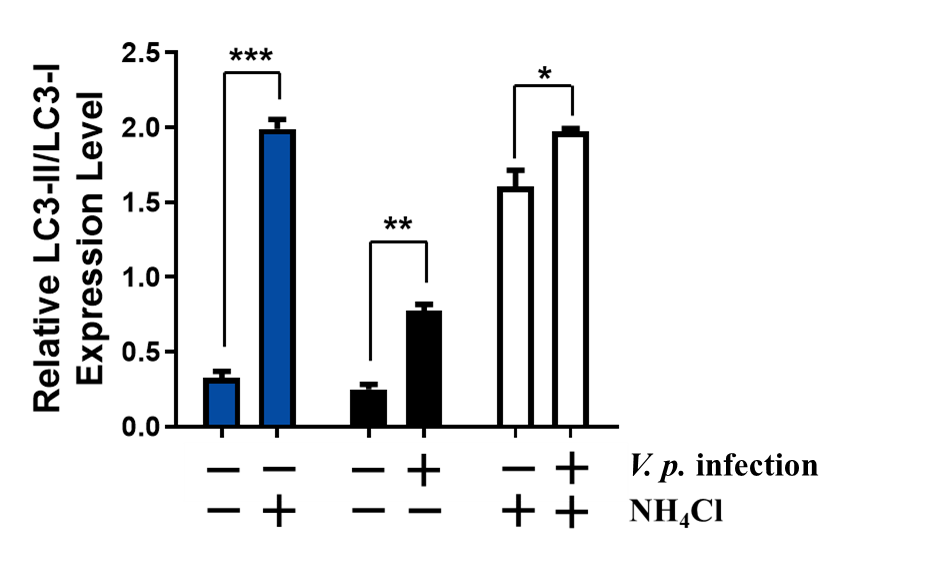


**Figure S7. Ratio of LC3-II to LC3-I in densitometric analysis by Student’s *t*-test between two groups.**

Data are presented as mean ± SEM (n = 3), with statistical significance determined at * *p* < 0.05, ***p* < 0.01, ****p* <0.001.

| Table S1. List of oyster genes targeted by real-time PCR | | |  |  |  |
| --- | --- | --- | --- | --- | --- |
| Gene name | Abbreviation | Primer sequence (5'-3') | Tm (℃)  (℃) | Product size (bp) | Efficiency (%) |
| Autophagy-related 10 | Atg10 | *f*: GAAGAGGGTGCCAGATGT | 55 | 232 | 97.7 |
|  |  | *r*: TTTTCCGTACTCCAGAGG |  |  |  |
| Autophagy-related 3 | Atg3 | *f*: ATACAGATGGTGGCAGACG | 56.5 | 304 | 96.7 |
|  |  | *r*: CAACGGTTACAGACAAGAGC |  |  |  |
| Autophagy-related 13 | Atg13 | *f*: AAAGGTTGGTTCTGTGCC | 55 | 166 | 103.1 |
|  |  | *r*: TTTGGGAGTAGTTGGTCG |  |  |  |
| Autophagy-related 101 | Atg101 | *f*: TGAATCGCCATGACTACG | 55 | 139 | 97.8 |
|  |  | *r*: CCAACAGATGACTGGGAAG |  |  |  |
| Autophagy-related 9a | Atg9a | *f*: AGAATGACACGCACAAGG | 55 | 185 | 95.5 |
|  |  | *r*: GGCAGTGAGATAAAAGGAGC |  |  |  |
| Autophagy-related 2 | Atg2 | *f*: TAGTCCTACCCCTCGTCA | 55 | 286 | 99.3 |
|  |  | *r*: CGTGGATGCTTGTTGTCA |  |  |  |
| Autophagy-related 4 | Atg4 | *f*: GACCCACATACTACGCAACT | 55 | 314 | 97.1 |
|  |  | *r*: TCTGAATCCTGTCCTCCC |  |  |  |
| Autophagy-related 1 | Atg1 | *f*: AGGCCTACAGTGCAGAGGAA | 56.5 | 244 | 101.8 |
|  |  | *r*: TTCCAAGGCTGCTGACTTTT |  |  |  |
| Autophagy-related 16 | Atg16 | *f*: CTGTTAGAGCAGCTTCGAC | 56.5 | 212 | 97.5 |
|  |  | *r*: TCCAGAGCCTGACTGTTTC |  |  |  |
| Autophagy-related 5 | Atg5 | *f*: TGGCATTATCCTGTTGGG | 55 | 178 | 102.2 |
|  |  | *r*: TGTGCTTCAGTGAGTCGG |  |  |  |
| Mechanistic target of rapamycin (serine/threonine kinase) | mTOR | *f*: GATGCTTCAGCGTGTCTT | 55 | 154 | 103.1 |
|  |  | *r*: CTTCTGTTGCTTGATGGC |  |  |  |
| Beclin 1 | Beclin 1 | *f*: TAACAAGCACCCCTGAAC | 55 | 113 | 100.5 |
|  |  | *r*: CAAAGCACTGGAAACTCG |  |  |  |
| Phosphatidylinositol 3-kinase, catalytic subunit type 3 | PIK3C3 | *f*: TCTCCAACTTTGAGCCATTA | 55 | 135 | 99.3 |
|  |  | *r*: CTCCACCTTCCACCATTT |  |  |  |
| Microtubule associated protein 1 light chain 3 | LC3 | *f*: TAACCCAGCGACTGAAAG | 55 | 198 | 96.0 |
|  |  | *r*: AAGCCTGCGAAGGATGAA |  |  |  |
| Glyceraldehyde 3-phosphate dehydrogenase | GAPDH | *f*: GGATTGGCGTGGTGGTAGAG | 55 | 184 | 100.1 |
|  |  | *r*: GTATGATGCCCCTTTGTTGAGTC |  |  |  |
